# A systematic approach to evaluate the influence of environmental conditions on eDNA detection success in aquatic ecosystems

**DOI:** 10.1371/journal.pone.0189119

**Published:** 2017-12-08

**Authors:** Bernhard C. Stoeckle, Sebastian Beggel, Alexander F. Cerwenka, Elena Motivans, Ralph Kuehn, Juergen Geist

**Affiliations:** 1 Aquatic Systems Biology Unit, Department of Ecology and Ecosystem Management, Technical University of Munich, Freising, Germany; 2 SNSB-Bavarian State Collection of Zoology (ZSM), Munich, Germany; 3 Unit of Molecular Zoology, Chair of Zoology, Department of Animal Sciences, Technical University of Munich, Freising, Germany; 4 Department of Fish, Wildlife and Conservation Ecology, New Mexico State University, Las Cruces, New Mexico, United States of America; University of Hyogo, JAPAN

## Abstract

The use of environmental DNA (eDNA) to determine the presence and distribution of aquatic organisms has become an important tool to monitor and investigate freshwater communities. The successful application of this method in the field, however, is dependent on the effectiveness of positive DNA verification, which is influenced by site-specific environmental parameters. Factors affecting eDNA concentrations in aquatic ecosystems include flow conditions, and the presence of substances that possess DNA-binding properties or inhibitory effects. In this study we investigated the influence of different environmental parameters on the detection success of eDNA using the invasive goby *Neogobius melanostomus*. In a standardized laboratory setup, different conditions of flow, sediment-properties, and fish density were compared, as well as different potential natural inhibitors such as algae, humic substances, and suspended sediment particles. The presence of sediment was mainly responsible for lower eDNA detection in the water samples, regardless of flow-through or standing water conditions and a delayed release of eDNA was detected in the presence of sediment. Humic substances had the highest inhibitory effect on eDNA detection followed by algae and siliceous sediment particles. The results of our study highlight that a successful application of eDNA methods in field surveys strongly depends on site-specific conditions, such as water flow conditions, sediment composition, and suspended particles. All these factors should be carefully considered when sampling, analyzing, and interpreting eDNA detection results.

## Introduction

The use of environmental DNA (eDNA) to determine the presence and distribution of aquatic organisms has become an important tool to investigate and monitor freshwater communities. This is particularly relevant for rare species and small populations of endangered species [1,2] as well as for early detection of invasive alien species (IAS) at the beginning of an invasion [3,4]. The widespread molecular method of eDNA takes advantage of a continuous DNA release of organisms into the environment [5,6], predominately from epidermal cells, excrement, hair, body fluids, and germ cells [7–10]. This unaffiliated DNA can later be extracted from the environment, i. e. from soil- or water-samples [11].

The detection of eDNA sensitively indicates the presence of a species in the environment. It has been used for decades, e.g. in studies targeting soil microbial diversity and ancient DNA [12]. Most recently, it has become a prominent tool for monitoring species and community compositions without direct intervention into the ecosystem [13]. An increasing amount of literature is available focusing on the development of suitable molecular markers, the improvement of sampling techniques, DNA extraction procedures, and PCR protocols [14,15]. However, environmental factors which may influence and change eDNA detection rates are still understudied [16]. There are only a few studies [16,17–19] targeting the effect of different environmental settings on eDNA persistence, the effects of water flow or distance between the locality of sampling and of the source organism, pH level, and degradation rates of eDNA on its detectability. In addition, most studies were not conducted under standardized laboratory conditions but in the field where several environmental factors may simultaneously affect results, hampering the identification and ranking of the importance of different factors.

eDNA may rapidly degrade [20,21] or even become undetectable, particularly in aquatic ecosystems with flowing water conditions [18]. In such environments, the maximal time of species detection has been reported to decrease to only few days [9] or even hours [18]. Thus, studies using eDNA should always consider environmental conditions and describe them explicitly. However, prior to that there is a strong need to investigate confounding factors which may potentially influence eDNA detection results. In this context, essential steps are to understand and isolate the effects of dilution in different aquatic environments (flowing and still waters) and also the effects of time-lag between species presence in an ecosystem and sampling implementation [9,22].

In addition to dilution processes in the water column, flow rate may also affect DNA persistence and thus eDNA detection rate [23,24] in aquatic environments, as well as solid materials and dissolved substances in the water column and the riverbed [11, 25]. It is assumed that the properties of sediments (suspended or benthic) may influence eDNA degradation [26]. The sediment may also adsorb DNA [20,21] and thus decrease detection rates. On the other hand, eDNA may be re-suspended from the sediment [27], leading to false positives, i.e. detection of a species that is not present in the environment any more [2]. In addition, dissolved substances in the water matrix may change DNA detection rates and even inhibit PCRs like humic acids [28]. Thus, knowledge on potential confounding factors is essential and should be considered when using eDNA in species detections. Inhibitory substances that influence the presence of free DNA in the water have to be considered separately from those inhibiting the DNA during further processing in the laboratory.

To date and to the best of our knowledge, no other study has yet systematically targeted cofounding and inhibiting factors for the application of eDNA in an aquatic species, under standardized laboratory settings. This is particularly alarming, since multiple studies address and stress the importance and the need for studies targeting environmental and physical-chemical factors.

The benthic invasive alien round goby *Neogobius melanostomus* (Teleostei: Gobiidae, Pallas, 1814) is an optimal model species in the context of this study, since it is a globally invasive species in which early detection is particularly important. Moreover, it is characterized by small home ranges, restricted mobility, small body size, little requirements for water quality, and it is well-studied [29,30], including studies on eDNA, i.e. [31]. The first species-specific primer pair for eDNA analysis was identified recently [31]. Nathan et al. [24] compared how well different PCR methods were able to detect round goby in experimental conditions. Our colleagues [32] optimized eDNA analysis to detect this species in riverine water samples and established a second primer pair specific to this species.

The objective of this study was to assess eDNA detection under different experimental conditions and to evaluate naturally occurring and potentially inhibiting factors in aquatic ecosystems. We specifically hypothesized that (i) fish density does not affect the success of eDNA detection, whereas (ii) the presence of sediment and (iii) humic substances (humus), (iv) water flow condition, and (v) longer time after a species had left an area decrease eDNA detection success.

## Material & methods

### Experimental design and sampling

For the experiments, 120 similarly sized round gobies (*N*. *melanostomus*) were caught by electrofishing in the upper Danube River (river-kilometer: 2,418, GPS: E 11°50’12”, N 48°54’01”) on November 19^th^, 2015 under license number 31–7562. They were maintained at the Chair of Aquatic Systems Biology (Technical University of Munich). In order to evaluate the influence of environmental conditions on eDNA detection, two experiments were implemented.

The collection was additionally approved by the local owner of the fisheries rights (Kreisfischereiverein Kelheim e.V.) and the state fisheries authority (Fischereifachberatung Niederbayern). Laboratory experiments were conducted according to German legislation (German Tierschutzgesetz, §11 TierSchG), license number 32–568), approved by the local veterinary board (Landratsamt Freising, license number 32–568) and the animal welfare committee at TUM. After the experiments were completed, the remaining gobies were maintained at the institute for teaching purposes. This study was conducted in compliance with the international animal care guidelines of the Association for the Study of Animal Behaviour and the ARRIVE guidelines.

#### Experiment 1: Impact of abiotic factors and fish density

In the first experiment, the impact of different abiotic factors (no flow, flow, sediment, no sediment) on the detection rate of round goby eDNA was tested. Additionally, the temporal change of detectability was verified by multiple sampling time points. In total, the experimental set-up consisted of twelve treatments with four controls without fish. Experimental trials were conducted under constant physicochemical conditions (mean ± SD: room temperature (T) = 12.3 ± 0.5°C, water temperature (T) = 12.8 ± 0.82°C, dissolved oxygen (DO) = 8.9 ± 1.2 mg L^-1^, electric conductivity (EC, at 25°C) = 1125 ± 5 μS cm^-1^, pH ~ 8, hardness = 482.14 ppm CaCO_3_) using local well water in new and unused plastic bins initially filled with 65 L of water. Light conditions were 12/12 h dark/light.

**Fig 1:**
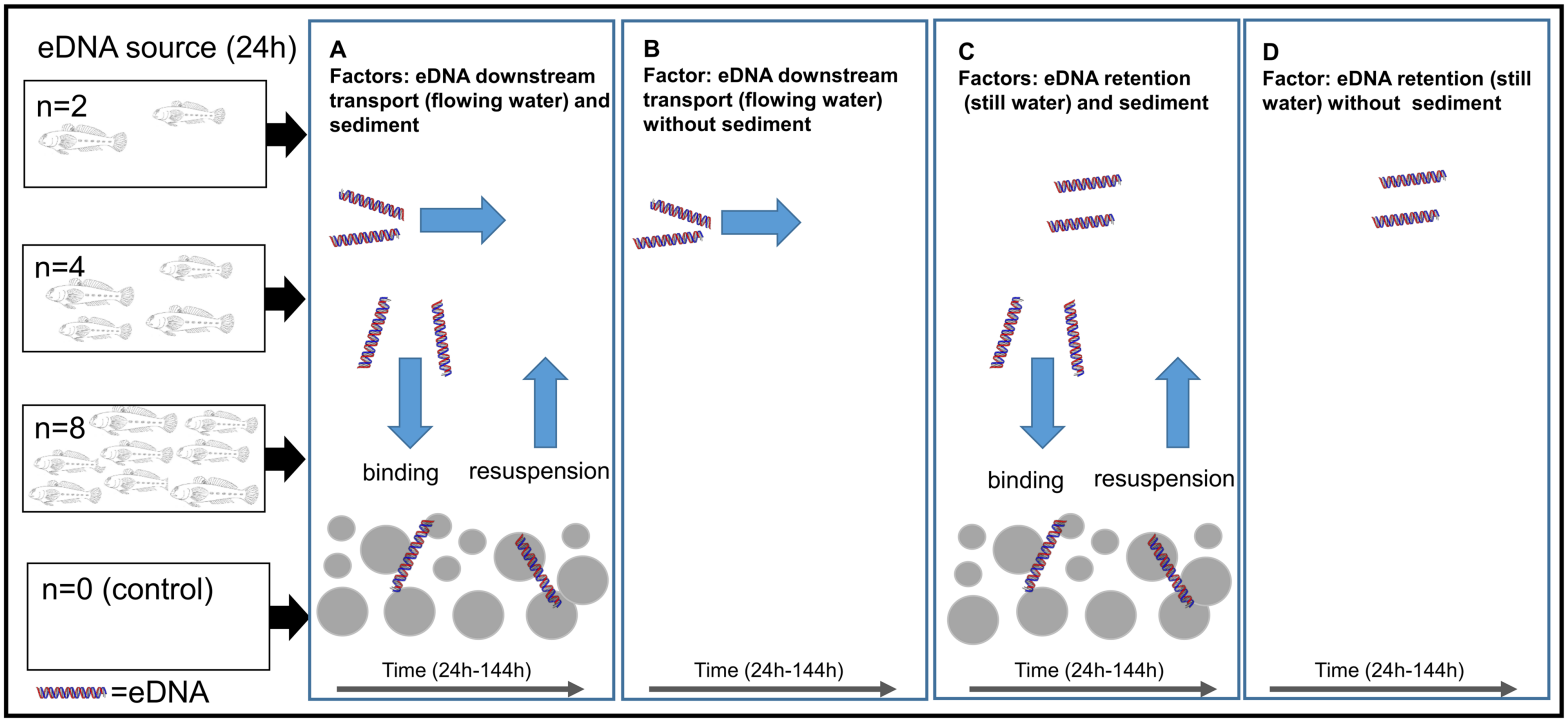
Conceptual diagram of the experimental design. The impact of different environmental processes (retention, transport, binding and resuspension) on eDNA analysis as a function of time and fish density (2, 4 and 8 fish): A: Treatment with sediment and flowing water, B: Treatment with flowing water without sediment, C: Treatment with still water and sediment, and D: Treatment with still water without sediment. Samples were taken after fish were removed (24 h after start of the experiment). In addition, the bins were sampled three times within 144 hours. A control without fish was included for every treatment.

The different combinations of abiotic and biotic conditions were: (1) sediment and flowing water (S-F), (2) no sediment and flowing water (NS-F), (3) sediment and no water flow (S-NF), and (4) no sediment and no water flow (NS-NF) (Fig 1A, 1B, 1C and 1D). All four condition combinations were tested with different goby densities, i.e. of two, four, and eight individuals per bin, plus one control without fish. The sediment, 1–2 mm quartz gravel aquarium sediment (Aqua Inspiration, Bonn, Germany), was washed before being placed in a 3 cm layer in the bins. All bins with flow-through conditions had the same water inflow and outflow (1.2 L min^-1^). For the experiments, specimens were randomly placed in 65 L bins according to the fish density of the specific treatment. Total fish biomass and round goby sex ratio did not differ significantly between S-F, NS-F, S-NF and NS-NF (both: Kruskal Wallis, df = 3, *P*-value > 0.1). Water samples for eDNA analyses were collected at five time points: (T0) before the fish were added (negative controls) and (T1) 24 hours after the fish were added (positive controls), samples were taken and the fish were removed. In addition, the bins were sampled (T2) 48 hours, (T3) 96 hours, and (T4) 144 hours after T0. Per time point, three water samples (500 mL each) were taken with sterile bottles (Thermo Scientific^™^ Nalgene^™^, Waltham, MA, USA) as biological replicates from each bin.

#### Experiment 2: Impact of inhibitors

In the second experiment, the impact of different potential natural inhibitors (algae (ALG), humus (HUM), and siliceous (SEDA) and calcareous (SEDB) fine sediment) on eDNA detection was tested. Initially, eight *N*. *melanostomus* were kept for 24 hours in a bin (still water and no sediment). After fish removal, 39 water samples were taken (500 mL each with sterile bottles). Each potential inhibitor was added to nine samples in concentrations of 10 mg L^-1^ (three samples), 100 mg L^-1^ (three samples), and 1000 mg L^-1^ (three samples).

The three untreated samples served as positive controls. In order to simulate the impact of algae which may interfere with the PCR reaction [33], the commercially available Shellfish Diet^®^ (Reed Mariculture, Campbell, USA), a mixture of *Nannochloropsis*, *Isochrysis*, *Pavlova*, *Thalassiosira*, and *Tetraselmis* algae, was used. The second inhibitor was natural HUM (Floragard, Oldenburg, Germany) which is frequently found in aquatic ecosystems [34]. SEDA is a grained commercial bentonite clay (Agrimont, Abensberg, Germany). This clay is abundant in the catchment of the river Danube in Southern Germany [35] and also occurs in other global locations (i.e. in America [36] and in Asia [37]). SEDB was grained limestone (CaCO_3_), a common surface bedrock, i.e. in the Southern and Northern Alps [38]. After the addition of the potential inhibitors, all samples were stirred for 10 seconds and then filtered within half an hour. Before filtering, pH and turbidity were measured. The observed values resembled those from natural waters [39] (treated samples: mean ± SD for turbidity: 9.0 ± 14.3 NTU; mean ± SD for pH: 8.4 ± 0.8; positive controls: mean ± SD for turbidity: 0.5 ± 0.02 NTU; mean ± SD for pH: 7.5 ± 0.1).

### Filtration and DNA extraction

All water samples were collected in the same way and filtered within half an hour after sampling using 0.4 μm glass fiber filters (Macherey-Nagel, Düren, Germany). As an extraction control three filters per each filtration session were soaked with deionized water. Filters were stored in sterile 2 ml tubes at -80°C until DNA extraction with the DNeasy Blood & Tissue Kit (Qiagen, Hilden, Germany).

### Primer design, PCR sensitivity and primer specificity

Primers were designed with Primer3 version 4.0.0 [40,41] based on a consensus sequence generated from all existing round goby Cytochrome Oxidase I (COI) sequences from the NCBI database (GenBank, www.ncbi.nlm.nih.gov, date of search 15^th^ of February 2016). To ensure species-specificity, primer sequences were compared to all available sequence data with BLAST (Basic Local Alignment Search Tool; Genbank, www.ncbi.nlm.nih.gov/blast, date of search 19^th^ of February 2016). The primers were NeoMel_NCOI1 5′- GGCCTCCTCTGGTGTTGAA-3′ (forward) and NeoMel_NCOI2 5′- GCCAGGTGAAGGGAGAAGAT -3′ (reverse) and amplify a 130 bp product. The annealing temperature for subsequent qPCRs was optimized in a gradient cycler (Mastercycler Gradient, Eppendorf, Germany) with *N*. *melanostomus* DNA, using 0.2 μm of each primer, 1.0 μL of PCR buffer, 1.0 μL of DNTPs, 1.2 μL of MgCl, 0.16 μL of Taq polymerase, and 4.2 μL of HPLC H_2_0, and 2 μl of DNA template.

In addition to the in silicio test for species-specificity, the specificity of the primers NeoMel_NCOI1 and NeoMel_NCOI2 was validated by qPCRs (protocol listed below) with archived DNA samples (Bavarian State Collection of Zoology; http://www.zsm.mwn.de/e/) from other goby species (*Proterorhinus seminularis*, *Ponticola kessleri*, *Babka gymnotrachelus*) occurring sympatrically in the Danube catchment area [42].

After primer optimization, NeoMel_NCOI1 and NeoMel_NCOI2 were compared by qPCR (protocol listed below) to the previously identified primers GobyCOI-F2 and GobyCOI-R2 [24] and SL_eDNA_NM_F1 and SL_eDNA_NM_R1 [32], for their sensitivity detecting *N*. *melanostomus* eDNA by using an eleven-fold DNA dilution series of 10 ng to 1 ag per μL (DNA template) as described previously [2]. The most sensitive primer pair detecting the lowest concentration was used for subsequent analyses of all water samples.

All water samples, the dilution series, and the species-specificity tests were analyzed by qPCRs according to a previous eDNA study [2]. The thermal profile was as follows: 95°C for 5 min (initial denaturation), 40 cycles with 95°C for 30 s (denaturation), 57°C (GobyCOI-F2/ GobyCOI-R2) and 60°C (SL_eDNA_NM_F1/ SL_eDNA_NM_R1 and NeoMel_NCOI1/ NeoMel_NCOI2) for 90 s (annealing) and 72°C for 30 s (extension and fluorescence acquisition), and 68°C for 10 min (final extension) with a final continuous fluorescence acquisition (65°C to 99°C) for the melting curve analysis. All products were visualized by gel-electrophoresis on a 1.8% agarose gel stained with SYBR^®^ Safe (Invitrogen, Karlsruhe, Germany).

In order to avoid contamination during filtering and PCR preparation, the same practice as described earlier [2] was applied. From each extracted water sample, three PCR replicates (technical replicates) were prepared following the qPCR protocol mentioned above. In each qPCR run, two reference samples (DNA templates with 1 pg per μl) were included in order to assess fluorescence signals and melting curves of extractions. PCR scoring (positive or negative) was performed according to PCR reactions were scored positive when an adequate melting curve occurred, the fluorescence signal was above the threshold (above signal of reference sample with 1 pg per μl DNA template) and a band of expected length was detectable on a 1.8% agarose gel stained with SYBR^®^ Safe (Invitrogen, Karlsruhe, Germany) [2].

### Statistical analysis

For both experiments, binary logistic regression models using SPSS v. 22 (IBM, USA) were used to evaluate the likelihood of positive DNA detection. The evaluation of the effect of each of the variables, on the positive PCR rate was done by assigning the PCR result (positive or negative) as a dependent variable with the following function:

f(positivePCR)=f(flow,sediment,fish,time)

where *f(positive PCR)* represents the PCR results (positive = 1; negative = 0) and *f (sediment*, *flow*, *fish*, *time)* refers to the explanatory variables (flow/no flow, sediment/no sediment, different fish densities, and time). The same analysis was repeated for each time point to test the changing magnitude of influence for the factors sediment, flow and fish density throughout the experimental period (T1, T2, T3 and T4).

In order to assess the influence of ALG, HUM, SEDA, and SEDB on the PCR result, a binary logistic regression was applied for each inhibitor individually with the following function:

f(positivePCR)=f(inhibitor)

where *f(positive PCR)* represents the PCR results (positive = 1; negative = 0) and *f(inhibitor)* refers to the potential inhibitors, ALG, HUM, SEDA, and SEDB. Models for the non-linear relationship between Cq values and inhibitor concentration were selected based on Akaike’s information criterion. Significance was accepted at α = 0.05.

## Results

### PCR sensitivity, species-specificity and quality control

The comparison of all three primer pairs showed that the newly designed primer pair, NeoMel_NCOI had the highest sensitivity and detected DNA down to 1 pg per μl DNA template of the dilution series (https://doi.org/10.5061/dryad.q609s). This primer pair was consequently used for subsequent analysis. The PCR test with other sympatric invasive alien goby species showed that the designed primer pair had the desired specificity, i.e. it only amplified DNA fragments of the target species. In line with the expectations, all water samples from treatments without fish, all control filters, and PCR controls did not result in positive goby eDNA signals.

#### Experiment 1: Impact of abiotic and biotic factors

**Fig 2:**
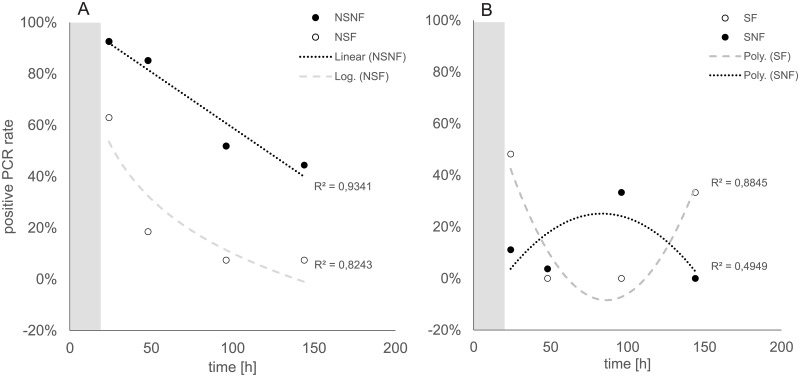
Positive eDNA detection rates over the 144 h experimental time course. Grey area: Duration of fish exposure. Fish were removed at time point 24 h. Sample size n = 27 per time point. A) Treatments without sediment (NS-NF: no sediment, no flow; NS-F: no sediment, flow) B) treatments with sediment (S-F: sediment, flow; S-NF; sediment, no flow).

All the experimental variables, i.e. presence of sediment and flow, and fish density, had a measurable influence on the eDNA detection rate. Over the full experimental duration (144 h), the strongest effect was observed for “sediment present” and “no flow” (S-NF). Here, goby DNA could only be verified in 12% of the water samples. In general, flowing water reduced the influence of sediment presence, with 20% of all samples testing positively for goby DNA with sediment (S-F) and 24% without sediment (NS-F) present. The highest overall species detection rates, 69%, were observed in treatments without flow and without sediment (NS-NF). Within the 144 h time course, we detected two distinct patterns in eDNA detection between treatments with and without sediment added (Fig 2A and 2B). In all treatments without sediment, the positive detection rate of goby DNA decreased continuously over time after the removal of the fish at time point 24 h, being more pronounced under flow conditions (Fig 2A). While the decrease is more linear without flow, the detection followed an exponential decay function for flow conditions.

In contrast, when sediment was present, a second peak of positive DNA detection at time point 96 h without flow and at time point 144 h under flow conditions was identified (Fig 2B), indicating a delayed presence of detectable fish DNA.

#### Binary logistic regression—Full model

The logistic regression model was statistically significant (χ^2^(14) = 181, *P* < 0.0001). The model explained 48.1% (Nagelkerke R^2^) of the variance in eDNA detection and correctly classified 80.1% of cases.

**Fig 3:**
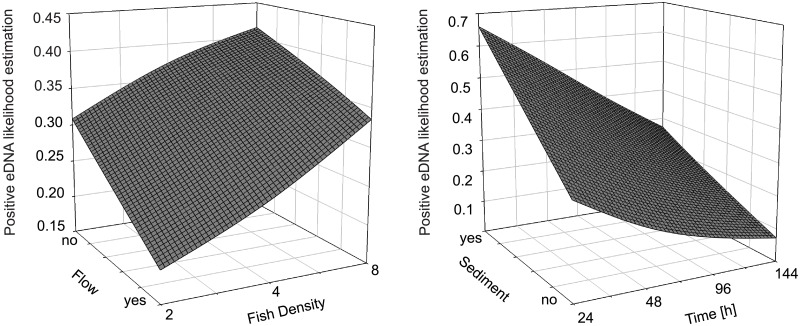
3D surface plots of significant 2-way interactions responsible for changes in positive eDNA detection likelihood.

The changes in one variable strongly depended on changes of the other variables. Fig 3 illustrates the different influence of the parameters on eDNA detection probability. There was a significant 4-way interaction between sediment presence, flow condition, fish, and time for the probability of positive eDNA detection (Table 1). Different combinations of explanatory variables lead to results that are significant, but show some increases and some decreases in detection, depending on treatment condition. For example, the interaction between fish*flow*sediment*time yields exp(B) to be 0.313, indicating a decrease in likelihood of detection (*P* < 0.001), but the interaction term between fish*flow*sediment is also significant (*P* = 0.014) with a very large increase in likelihood (exp(B) = 18.732), indicating varying processes of eDNA transport and resuspension over time. All other underlying 3-way interactions were also significant, being most pronounced between fish density, flow condition, and time (*P* = 0.002). Significant 2-way interactions were observed for sediment presence and time (*P* = 0.044) and fish density and flow condition (*P* = 0.024). The latter interaction fish*flow yields a significant decrease in likelihood of detection (*P* value = 0.024, exp(B) = 0.013), representing strong dilution effects as illustrated in Fig 3.

**Table 1:** Full model results for binary logistic regression. Likelihood estimation for positive eDNA detection for different experimental conditions and variable interactions for sediment presence (0/1), flow condition (0/1), fish density (n = 2,4,8) and time point (t = 0,24,48,144h).

Variables	B	Standard error	Wald	df	*P*—value	Exp(B)	95% confidence interval for EXP(B)	
							lower	upper
Sediment	2.688	3.086	0.759	1	0.384	14.707	0.035	6231.262
Flow	2.573	3.239	0.631	1	0.427	13.109	0.023	7484.979
Fish	1.933	2.116	0.834	1	0.361	6.907	0.109	436.633
Time	-0.228	1.539	0.022	1	0.883	0.796	0.039	16.277
Fish * Flow * Sediment * Time	-1.162	0.311	13.952	1	**<0.001**	0.313	0.17	0.576
Fish * Flow *Sediment	2.93	1.192	6.045	1	**0.014**	18.732	1.812	193.67
Fish * Sediment * Time	1.083	0.474	5.227	1	**0.022**	2.953	1.167	7.469
Flow * Sediment * Time	2.411	0.91	7.029	1	**0.008**	11.15	1.875	66.304
Fish * Flow * Time	1.136	0.375	9.155	1	**0.002**	3.114	1.492	6.501
Flow * Sediment	-2.151	2.203	0.953	1	0.329	0.116	0.002	8.726
Fish * Sediment	-1.985	1.403	2.001	1	0.157	0.137	0.009	2.15
Sediment * Time	-3.456	1.716	4.056	1	**0.044**	0.032	0.001	0.911
Fish * Flow	-4.375	1.934	5.116	1	**0.024**	0.013	0	0.558
Flow * Time	-1.543	1.003	2.367	1	0.124	0.214	0.03	1.526
Constant term	-0.267	4.167	0.004	1	0.949	0.765		

“*” denotes an interaction.

Significant *P*—values are indicated in bold.

#### Fish exposure time course

Logistic regression models were additionally calculated for each time point after the removal of the fish to gain additional information about the influence of the factors sediment, fish and flow within the time course (Table 2). The results show that, depending on the time point the sample was taken, the parameters sediment, flow and fish density had changing influence on the overall detection of eDNA. Initially (24 h), the presence of sediment had the highest influence on positive DNA detection likelihood (*P* < 0.001) while fish density had no significant effect on detectability (*P* = 0.589). At time points 48 h and 96 h, the flow condition played a major role for detection after removal of the fish. Positive eDNA detection likelihood was highest at time point 96 h. At the last time point (144 h), the initial fish density was the only factor with a significant influence on eDNA detection (*P* = 0.012) since a presumable delayed release of sediment bound DNA seems dependent on this parameter. An increase in fish density by the factor 2 increased positive detection likelihood by 124%.

**Table 2:** Binary logistic regression analysis during the 144 h experimental time course. Likelihood estimation for positive eDNA detection for different experimental conditions sediment (0/1), flow (0/1) and fish density (n = 2,4,8). Significant *P*—values are indicated in bold.

Time point	Variables	B	Standard error	Wald	df	*P*—value	Exp(B)	95% confidence interval for EXP(B)	
								lower	upper
24 h	Sediment	2.129	0.445	22.906	1	**<0.001**	8.408	3.516	20.108
	Flow	-0.195	0.442	0.195	1	0.659	0.823	0.346	1.958
	Fish	-0.146	0.271	0.291	1	0.589	0.864	0.508	1.470
	Constant term	-2.414	1.061	5.178	1	**0.023**	0.089		
48 h	Sediment	5.556	1.256	19.558	1	**<0.001**	258.742	22.056	3035.336
	Flow	3.673	0.862	18.142	1	**<0.001**	39.362	7.263	213.337
	Fish	0.998	0.500	3.984	1	**0.046**	2.712	1.018	7.226
	Constant term	-18.471	3.800	23.629	1	**<0.001**	0.000		
96 h	Sediment	1.006	0.553	3.312	1	0.069	2.735	0.925	8.084
	Flow	3.16	0.799	15.648	1	**<0.001**	23.574	4.925	112.83
	Fish	0.649	0.342	3.6	1	0.058	1.914	0.979	3.743
	Constant term	-9.464	2.087	20.563	1	**<0.001**	0		
144 h	Sediment	0.598	0.496	1.455	1	0.228	1.819	0.688	5
	Flow	0.120	0.490	0.06	1	0.807	1.127	0.432	2.945
	Fish	0.809	0.321	6.345	1	**0.012**	2.246	1.197	4.215
	Constant term	-4.149	1.360	9.305	1	**0.002**	0.016		

### Experiment 2—Inhibitors

Each of the experimental treatments (algae (ALG), humus (HUM), siliceous (SEDA) and calcareous (SEDB) fine sediment) caused a different effect on eDNA detection success (positive PCR rate): 41% for HUM, 59.2% for ALG and 62.9% for SEDA, while 100% for SEDB (https://doi.org/10.5061/dryad.q609s).

**Fig 4:**
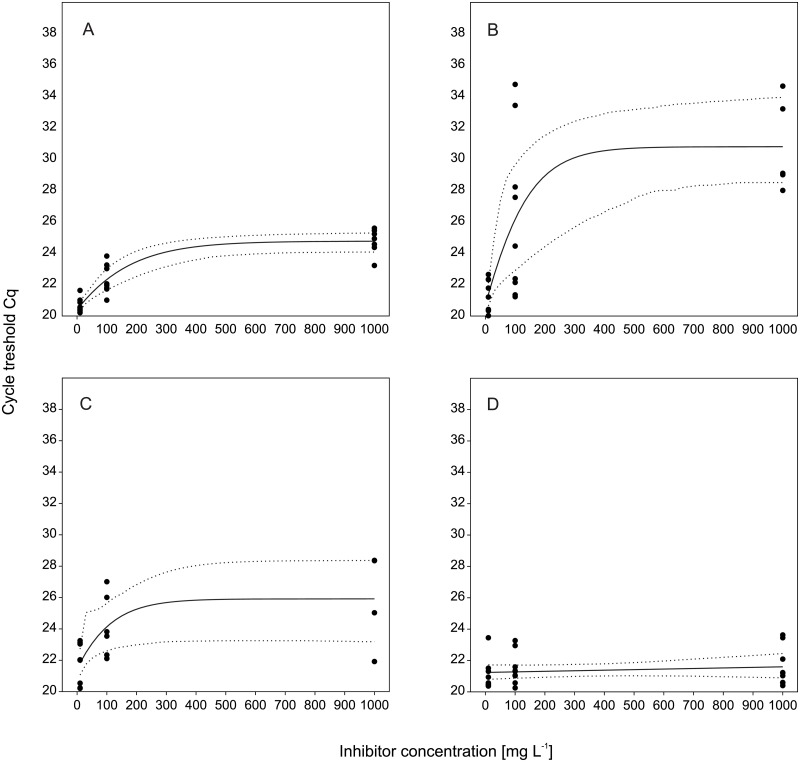
DNA quantification with different inhibitors. Cycle thresholds of goby DNA quantification with different inhibitors at 10, 100, and 1000 mg L^-1^. Cq values are fitted to a logarithmic trend. Higher Cq values represent a lower amount of DNA. A) algae, B) humus, C) a sediment, D) b sediment. Dotted lines indicate 95% confidence limits.

Inhibitory magnitude showed to be concentration-dependent for ALG, HUM and SEDA up to 1000 mg L^-1^, but not for SEDB (Fig 4). According to the binary logistic regression analysis, the increase of inhibitor concentration had a significant influence on positive eDNA detection likelihood with a decrease of 91.1% for HUM (*P* = 0.001), 78% for ALG (*P* = 0.004), and 72.2% for SEDA (*P* = 0.009) (Table 3). The calculation for SEDB was not possible since there was no relationship between concentration of SEDB and eDNA detection.

**Table 3:** Binary logistic regression analysis for different inhibitor effects (algae (ALG), humus (HUM), siliceous (SEDA) and calcareous (SEDB) fine sediment) on positive eDNA detection. Significant *P*–values are indicated in bold.

Variables	B	Standard error	Wald	df	*P*—value	Exp(B)	95% confidence interval for EXP(B)	
							lower	upper
ALG	-1.512	0.527	8.245	1.000	**0.004**	0.220	0.079	0.619
Constant term	3.587	1.187	9.130	1.000	**0.003**	36.140		
HUM	-2.414	0.752	10.302	1	**0.001**	0.089	0.020	0.391
Constant term	4.168	1.396	8.915	1.000	**<0.001**	64.591		
SEDA	-1.281	0.487	6.908	1.000	**0.009**	0.278	0.107	0.722
Constant term	3.296	1.112	8.782	1.000	**<0.001**	27.011		
SEDB	-	-	-	-	-	-	-	-
Constant term	-	-	-	-	-	-		

## Discussion

Based on a standardized laboratory setup, this study demonstrated and systematically quantified the effects of abiotic (sediment presence, flow condition, time) and biotic factors (fish density) on the eDNA detectability of an aquatic species, and the inhibitory effects of particulate matter and humic substances.

### Impact of abiotic and biotic factors

Our results show that the detection probability of eDNA generally increases with specimen density, but the detection is also influenced by the sediment and flow conditions as well as the time point when the sample is taken. Effects of dilution, sediment binding and re-suspension have a strong influence on the detection, depending on experimental conditions. Thus, our findings do not fully contradict previous experimental studies where no or little effects of density on detection rates were found ([23,24], but see [43]). However, according to Takahara et al. [44], the DNA release into the environment is correlated to the source organism biomass and not to their density. Also, the strength of this effect is likely linked to the dilution range determined by the fish biomass per water volume as well as to activity. To date, several studies tried to use eDNA quantity to infer species biomass, e.g. [18,44,45]. Nonetheless, results in the literature remain controversial and quantitative eDNA data with respect to density or biomass estimation should be interpreted cautiously since results may additionally be affected by other environmental factors such as flow condition or sediment type in the waterbody.

According to our experiments, presence of fine sediment decreases the success of eDNA detection, above all, when organisms start to inhabit an environment. DNA is secreted into the water body, resulting in detectability soon after first occurrence, and a subsequent accumulation over time. This delayed increase of DNA in the water might result when the maximal DNA binding capacity of the specific sediment is exceeded [21], likely due to saturation of non-covalent associations between the nucleic acids [20]. This result indicates that eDNA may be detected even after a species has become extinct or is removed from the ecosystem, as DNA may be retained by the sediment [46]. In this context, a previously implemented study [22] found that DNA persisted in the sediment for up to 132 days after the removal of bigheaded Asian carp *Hypophthalmichthys nobilis* from the ecosystem. It was previously shown [47] that sediment can preserve DNA in aquatic sediments even for thousands to hundreds of thousands of years. In contrast, a marine study found that the DNA degradation was higher in sediments, decreasing the maximal time of species detection [48]. Thus, detailed specification and information of the sampling locality and the corresponding environmental conditions are essential when using eDNA. This is particularly true, since species specific behavioral adaptations may increase or decrease detectability. In the case of *Neogobius melanostomus*, the species may (i) dig itself into the sediment and (ii) reduce its activity (pers. observation, [49]). Thus, the presence of sediment may result in false negatives or false positives and must be considered in studies using eDNA.

As documented in our study, flow current of water bodies also interferes the eDNA detection success which matches previous findings [11]. DNA is transported horizontally (e.g. by flowing water) and vertically (sinking effect) in aquatic environments [1]. This may affect species detectability applying the eDNA technique: flow may rapidly transport and dilute eDNA, and thus reduce the success of detection at the source location [1] and sedimentation effects may decrease the concentration of free DNA by binding and precipitation. In this context, reophilic fish species were detected up to 240 m downstream from the place were the species occurred, when using eDNA [46]. Similarly, Jane et al. [16] found positive eDNA counts to decrease with increasing flow speed. However, the same authors did not identify significant differences in detection rates when pooling samples of flowing water and still water. In our laboratory experiments, flow decreased eDNA detection 48 hours after the fish were removed from initially 63% to 7% (NS-F) and from 48% to 0% (S-F). In addition, feces can be a significant source of DNA. Fish feces eventually sink to the bottom which results in a conglomeration of DNA in sediments [50]. These effects may apply over a longer time period than the investigated one (six days) of this study and need further examination. However, DNA degradation starts immediately after eDNA is shed due to limited chemical stability [9]. eDNA of common carp *Cyprinus carpio* was detected with a probability of less than 5% after 4 days [26] whereas European flounder *Platichthys flesus* was not detected after 0.9 days probably due to DNA degradation [1]. In sum, for the time period of eDNA sampling several potential effects are to be considered when conducting eDNA research: potential DNA-transportation (vertical and horizontal), effects of dilution, retention, inhibition, and degradation. In our experiment, the greatest detection rates occurred directly after removal of the specimen from the bins and declined sharply thereafter.

Each of the environmental variables (flow condition, sediment presence, and specimen density) investigated in this study cannot be interpreted regarding their influence on the eDNA detection rate in an isolated way since there are significant interactions within these variables and over the time course. In addition, individual and species-specific behaviors may influence eDNA detection rates, e.g. known from mollusks or burrowing animals [51, 52]. Lastly, eDNA may interact with (dissolved) particles and may (re-)suspend from or to the sediment in aquatic environments.

### Inhibitors

Natural occurring inhibitory substances are a widely neglected topic in eDNA studies. However, such inhibitors may be omnipresent in aquatic ecosystems and can even be concentrated when water samples are filtered. This is particularly important, since the PCR is an enzymatic reaction and it is highly sensitive to inhibition [53].

The inhibitory effects of humus on PCR reactions have been well documented [54,55]. Humic acid is the most potent component of environmental humus, and inhibits PCR even at low concentrations. It binds to the template DNA, inhibits the amplification during PCR [53] and blocks the DNA polymerase [56]. In addition, it inhibits detection by quenching the fluorescent signal of SYBR Green, possibly preventing the dye from binding to DNA [57].

To our knowledge there are no previous studies on inhibiting effects of calcareous and siliceous sediments on eDNA detection. This is striking, since it was reported earlier [53] that calcium likely acts as a competitive inhibitor to magnesium, which is a cofactor for the polymerase enzyme and consequently calcium compounds reduce amplification efficiency. In the present study, the calcareous (SEDB) sediment exhibited no inhibition, whereas the siliceous sediment (SEDA) showed inhibitory effects but to lesser extent than algae and humic substances.

In addition to the inhibitory effects of humus detected in this study, the presence of algae also decreased detectability of eDNA. A possible explanation is that aquatic and terrestrial plants and algae contain substances that may interfere with the PCR reaction [33]. In particular, macroalgae contain acids, polysaccharides and phenols [58], which can be strong enzyme inhibitors. However, the inhibitory effect observed in this study could also be a result of the high concentration of non-target algal DNA, which may interfere and mask the detection of target DNA [59].

So far and predominantly, the demand for the standardization of eDNA studies is limited to laboratory work (i.e. PCR) and sampling protocols, e.g. [6]. With respect to future standardization approaches and in order to improve the applicability of this technique for routine monitoring and inventory approaches, it would also be essential to consider and define environmental factors which may influence results. This is particularly important to increase comparability of studies, especially if they are conducted in different environments or under different ecological conditions. Additionally, eDNA sampling should always be tailored to the species, e.g. sampling should be conducted near the substrate for benthic or bottom dwelling aquatic species [32]. Lastly, when sampling is conducted in lotic ecosystems, multiple samplings at different locations and time points are required, especially to confirm that a species is or has been present [60].

PCR inhibitors occur in almost every aquatic ecosystem. To avoid false negative results when conducting eDNA studies, potential inhibitors should be identified before starting an analysis. One simple method to exclude inhibition is to force an internal positive control by adding the targeted DNA to selected samples [60]. Once inhibition has been detected, several mitigation methods can be tested, like bovine serum albumin (BSA), inhibitor resistant polymerases, and dilution of samples.

### Conclusions

The application of eDNA method is popular, useful, sensitive, temporally accurate, and widespread in numerous biological fields. However, as demonstrated by the results of this study, there are many factors that can influence eDNA detection in a different way and are highly interdependent. The combination of single environmental factors may cause both false positives and false negatives. In addition, the transportation of DNA (vertical and horizontal) may falsify or change results. Thus, when applying this method, basic standard requirements need to be fulfilled and results must be carefully interpreted and results should always be placed in the context of the prevailing environmental conditions in the sampled habitat. Therefore, detailed information on the sampling, the species, the ecosystem, as well as potential inhibiting substances interfering with eDNA, should be routinely reported in every study.
